# Pulmonary artery embolism by a metal fragment after a booby trap explosion in a combat patient injured in the armed conflict in East Ukraine: a case report and review of the literature

**DOI:** 10.1186/s13256-018-1834-5

**Published:** 2018-11-05

**Authors:** Igor Khomenko, Ievgen Tsema, Pavlo Shklyarevych, Kyrylo Kravchenko, Victoriia Holinko, Sofiia Nikolaienko, Sergey Shypilov, Oleg Gerasimenko, Andrii Dinets, Vladimir Mishalov

**Affiliations:** 1 Department of Abdominal Surgery, National Military Medical Clinical Center of Ministry of Defense of Ukraine, Kiev, Ukraine; 2 Department of Surgery #4, Olexandrivska Teaching Hospital, Bogomolets National Medical University, Shovkovychna 39/1 str, Kiev, Ukraine; 3 Department of Thoracic Surgery, National Military Medical Clinical Center of Ministry of Defense of Ukraine, Kiev, Ukraine; 4 Department of Thoraco-Abdominal Surgery, Military Medical Clinical Center of the Northern Region of Ministry of Defense of Ukraine, Kharkiv, Ukraine; 5 Department of Abdominal Surgery, Military Medical Clinical Center of the Sothern Region of Ministry of Defense of Ukraine, Odessa, Ukraine

**Keywords:** Projectile-embolus, Combat trauma, Damage control, Hybrid warfare, Armed conflict in Ukraine

## Abstract

**Background:**

Pulmonary artery embolization due to projectile embolus is a rare complication in combat patients. Such embolization is rare for combat patients in the ongoing armed conflict, in East Ukraine since 2014.

**Case presentation:**

We report a clinical case of a 34-year-old Caucasian combat patient who was injured after an explosion of a booby trap hand grenade. This soldier was diagnosed with severe abdominal and skeletal trauma: damage of the duodenum and transverse colon, internal bleeding due to inferior vena cava damage and fractures of both lower extremities. The patient was treated at a highly specialized surgical center within the “golden hour” time. Whole-body computed tomography scan was performed as a routine screening method for hemodynamically stable patients, at which we identified a projectile embolus due to the explosion of a booby trap hand grenade in the right midlobar pulmonary artery. Our patient had no clinical manifestation of pulmonary artery embolism. At follow-up, our patient was diagnosed with the following complications: multiple necrosis and perforations of the transverse colon leading to a fecal peritonitis; duodenum suture line leakage caused the formation of a duodenal fistula; postoperative wound infection. These complications required multiple secondary operations, and in accordance to the principles of damage-control tactics, the extraction of projectile-embolus was postponed. Open surgery retrieval of the metal fragment was successfully performed on the 80th day after injury. Our patient was discharged from the hospital on day 168th after injury.

**Conclusions:**

Literature analysis shows a significant difference of clinical management for patient with projectile embolism in hybrid war settings as compared to previously described cases of combat and civil gunshot injuries. Damage control tactics and the concept of the “golden hour” are highly effective for those injured in a hybrid war.

A whole-body computed tomography scan is an effective screening method for asymptomatic patients with projectile-embolism of the great vessels.

The investigation of a greater cohort of combat patients with severe injuries and projectile-embolism should be performed in order to develop a better guideline for these patients and to save more lives.

## Background

The armed conflict in certain districts of Donetsk and Luhansk regions of Ukraine has been ongoing since 2014 [1–3]. Local and international communities considered it to be as a “hybrid war”, which was extensively studied and was defined as “the warfare with opponent forces who simultaneously and adaptively apply conventional weapon and irregular tactics, terrorism and criminal elements in the fight” [4]. Various types of high-energy weapons are applied against the Armed Forces of Ukraine such as artillery, multiple launch rocket systems as well as land mines and improvised explosive devices (IEDs). The application of high-energy weapons is associated with severe injury of the military personnel, which frequently results with thoracoabdominal trauma or extremity amputations [5].

According to previously published case report studies, arterial or venous embolism due to bullet or fragments from explosive devices is a rare complication of combat injuries. [6–8]. IED fragment embolism may be the cause of death due to its association with limb-threatening ischemia, sepsis, endocarditis, cardiac valves insufficiency, pulmonary embolism, and stroke [9]. Furthermore, the arterial embolism due to migration of the metal fragments is а diagnostic challenge for the military surgeons because of rare presentation and difficulties in diagnosis.

In recent literature, X-ray and computed tomography (CT) were used for detecting the bullet emboli in the blood vessels of patients with gunshot injuries in non-war cases. Still, such kind of embolism is а rare condition and it may be suspected in patients with perforating injuries, bleeding into the peritoneal, or retroperitoneal spaces. Nevertheless, the possibility of bullet or metal fragment embolism cannot be ignored [10, 11]. Previously published reports of bullet-embolism suggested following diagnostic features of the high-risk possibility for projectile embolism from projectile fragments: changing of shrapnel’s position in the subsequent X-ray images, that is, “roaming bullet” phenomenon; absence of the exit wound [12]; the number of entrance wounds is higher than the number of exit wounds [7]; the actual location of the embolus metal fragment according to X-ray or CT data does not correspond to the location of the bullet wound channel [6, 8–10, 13]. If the above-mentioned diagnostic signs are detected in patients after a gunshot injury, a whole-body CT scan and angiography is indicated to clarify the localization of the projectile embolus and to evaluate the distal circulation [6, 7, 12].

Important features of war surgery such as the “golden hour” principle and the damage-control tactics were also introduced in the present case report. We followed the principle of the “golden hour”, aiming to reduce time between injury and medical care and to increase the rate of saved lives in combat patients [1]. Damage control tactics are frequently applied in modern armed conflicts. This is a clinical approach in military medicine aiming to control bleeding and the microbial contamination with the combinations of surgical interventions and resuscitation: intraperitoneal or thoracic packing and rapid closure of wounds followed by resuscitation to correct fluid homeostasis, and to control coagulopathy with subsequent application of re-exploration [1, 14, 15].

This study to report a rare clinical case of a combat patient who was diagnosed with pulmonary artery embolism due to migration of metal fragment after explosion in area of hybrid war in Donbas, East Ukraine. This case report also demonstrated the utility for the application of damage control tactics in settings of ongoing hybrid armed conflict as well as the utility of the application of whole-body CT scan for all combat patients injured due to high-energy weapon application. In addition, this case report summarizes other published series in order to compare other clinical courses and the management of clinical cases of patients with foreign body embolism.

The aim of this study was to report a clinical case of a combat patient who was injured in the “hybrid war” in East Ukraine. The patient underwent a whole-body CT scan and was diagnosed with an embolus of the pulmonary artery due to fragment migration after an explosion of a booby trap hand grenade. The other goal of this report was to review existing data from previously published articles in order to summarize clinical features of blood vessel embolism by projectile fragment due to the gunshot injuries or high-energy explosions.

## Case presentation

A 34-year-old Caucasian man was injured due to an explosion in May 2017 at a combat mission in East Ukraine (Donetsk region). Our patient’s status was critical at admission. He was diagnosed with a penetrating blind wound of the abdomen, penetrating damage of the duodenum, colon, and marginal damage of the inferior vena cava, and acute hemorrhage. As a result of shelling after the booby trap explosion, our patient presented with wounds and fractures of both lower extremities. Our patient was in sopor at admission; other neurological parameters were not evaluated. Parameters of his vital functions (e.g. heart rate, blood pressure, oxygen saturations, etc.) were unknown at the time of admission, because they were not included in the official medical records at the first medical aid stage at the battlefield.

First medical aid was provided immediately at the place of injury by medical military personnel: a tourniquet was placed on his right lower extremity; intramuscular injection of 2 mg of butorphanol, 100 mg of doxycycline, 0.5 ml of anatoxin against *Clostridium tetani*. Both lower extremities were immobilized by using the ladder splint. Subsequently, our patient was urgently evacuated by ambulance to the nearest hospital and was provided with qualified medical aid.

Qualified surgical aid was provided at the Central District Hospital of Toretsk City. Our patient was admitted to the hospital within 55 minutes of his injury, according to the principle of the “golden hour”. Antibiotic therapy was continued with administration of doxycycline, 100 mg. He was diagnosed with a penetrating wound of the abdominal wall (the dressing on the abdomen was impregnated with intestinal contents and dark blood) and severe hemorrhagic shock due to continuing internal bleeding. Our patient underwent urgent laparotomy. During the operation we detected such injuries as: a perforating missile wound of the transverse colon with torn edges, a perforating missile wound of the horizontal part of the duodenum (the third part), a focus of primary traumatic necrosis at the head of the pancreas, diffuse fecal peritonitis in reactive phase. There was approximately 1800 mL of blood in the retroperitoneal space, hence a large and tense hematoma was diagnosed. The hematoma was aspirated, and further revision of the retroperitoneal hematoma site revealed the source of bleeding, which was a marginal wound of the infrarenal part of the inferior vena cava. Vascular suturing was performed to achieve persistent hemostasis. Colloid and crystalloid solutions, along with blood units were infused to maintain arterial blood pressure. To treat hemorrhagic shock our patient underwent resuscitation by transfusion of three doses of 270 mL of red cell mass, 2 doses of 250 mL of fresh frozen plasma, infusion of colloid and crystalloid solutions. Taking into account the critical status of the patient, the decision was made to minimize surgical interventions of the intestinal injuries according to the principles of damage-control tactics. Thus, intestinal wounds were sutured and a nasojejunal intubation was performed. Resection of necrotic focus at the head of the pancreas was not carried out. In order to stabilize systemic hemodynamics, after abdominal wall closure a 30-minute intraoperative pause was taken to continue intensive infusion therapy. Then we began surgical interventions to treat the extremity wounds. After tourniquet removal, bleeding from the right tibial artery was revealed without signs of critical limb ischemia. To stop the bleeding, ligation of the right posterior tibial artery was performed. Fractures of the left tibia were treated by extrafocal osteosynthesis using a rod external fixation device at the stage of specialized surgical aid on May 31, 2017 (second day after injury).

Specialized surgical aid was provided at the Military Medical Clinical Center of the North Region in Kharkiv. Upon admission, our patient underwent ultrasonography (US) of the abdominal and pleural cavities to check for possible complications. Further treatment modalities at the stage of specialized surgical aid included wound redressing, infusion therapy by colloids and crystalloids, ceftriaxone 4 g/day, metronidazole 1 g/day, parenteral nutrition by protein-based and medium chain triglycerides mixture, pantoprazole 40 mg/day, metoclopramide hydrochloride 20 mg/day, drotaverine hydrochloride 80 mg/day, enoxaparin sodium 80 mg/day, ferric(III)-hydroxide polyisomaltosate 200 mg/day), two transfusions of red blood cells and fresh-frozen plasma, albumin 20 g/day, infusion and vitamins (B1, B12), physiotherapy. Thoracic X-ray was not performed, since a whole-body CT scan is routinely performed for all hemodynamically stable patients with projectile and blind gunshot wounds according to the protocol of Military Medical Department of Ministry of Defense of Ukraine beginning July 2014.

**Fig. 1:**
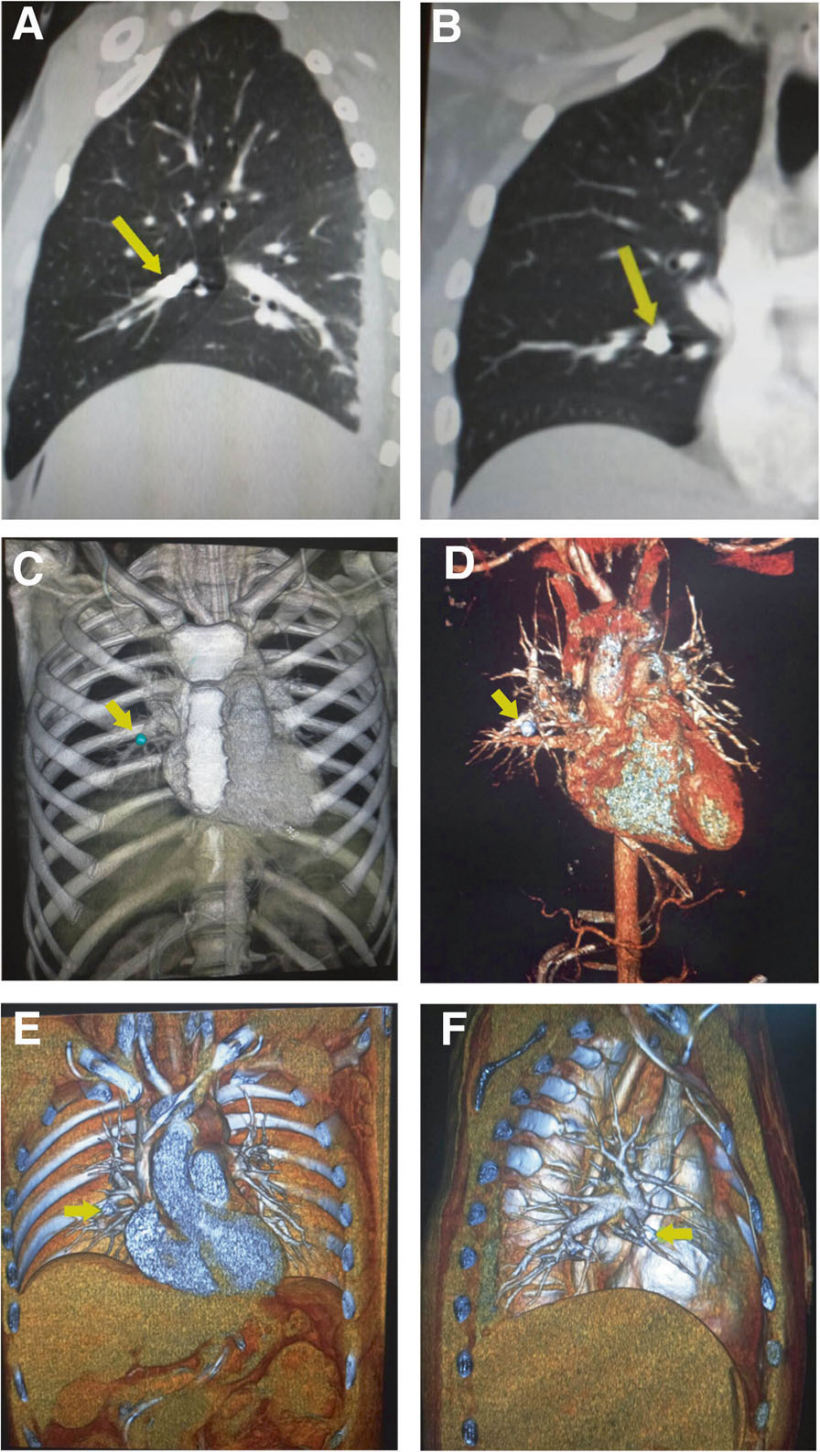
Visualization of the metal fragment in the branch of the right mid-lobe pulmonary artery by spiral computed tomography (CT) scan on the third day after the injury. **a** A photograph of the two-dimensional reconstruction of the CT frontal view image with the projectile embolus in the right pulmonary artery (*marked with an arrow*); **b** A photograph of the two-dimensional reconstruction of the CT sagittal view image with the projectile embolus in the branch of the right mid-lobe pulmonary artery (*marked with an arrow*) (**c**) A photograph of three-dimensional reconstruction of the CT front view image showing the pulmonary artery with the projectile embolus (*marked with an arrow*); **d** A photograph of CT angiography illustrating the projectile embolus (*marked with an arrow*); **e** A photograph of three-dimensional reconstruction of the CT angiography illustrating projectile embolus (*marked with an arrow*); **f** A photograph of three-dimensional reconstruction of the CT angiography showing projectile embolus in the pulmonary artery (*marked with an arrow*)

The US scan showed no dilatation of the inferior vena cava. Blood flow was retained, and a small volume of fluid in the right pararenal fatty tissue was identified. To our surprise, CT analyses revealed a foreign body of metallic density of 9.5 × 6.5 mm in the middle lobe branch of the right pulmonary artery (Fig. 1). Considering the absence of entrance wounds at the chest, a migration of the metal fragment was suspected from the inferior vena cava at the time of suturing, considering the embolus type of the foreign body. Severe trauma and damage control tactics were taken into consideration, thus we decided not to remove the metal fragment immediately, but to postpone its removal until after stabilization of his abdomino-skeletal injuries.

Our patient started enteral feeding through nasojejunal tube on June 1, 2017 (the third day after his injury). Drainage tubes from the site of the inferior vena cava and pelvic region were removed, one at the horizontal part of the duodenum remained. On June 6, 2017 (the eighth day after his injury) a bile leakage was detected from the remaining drainage tube and a contrast CT scan of his abdomen was performed. Analyses of the CT scan showed accumulation of contrast in the front of the descending part of the duodenum within an area of 6 × 5 × 6 mm without clear boundaries; in the same area a limited fluid cluster of 32 × 38 × 34 mm was identified with air bubbles and without clear boundaries, suggesting an intra-abdominal abscess.

**Fig. 2:**
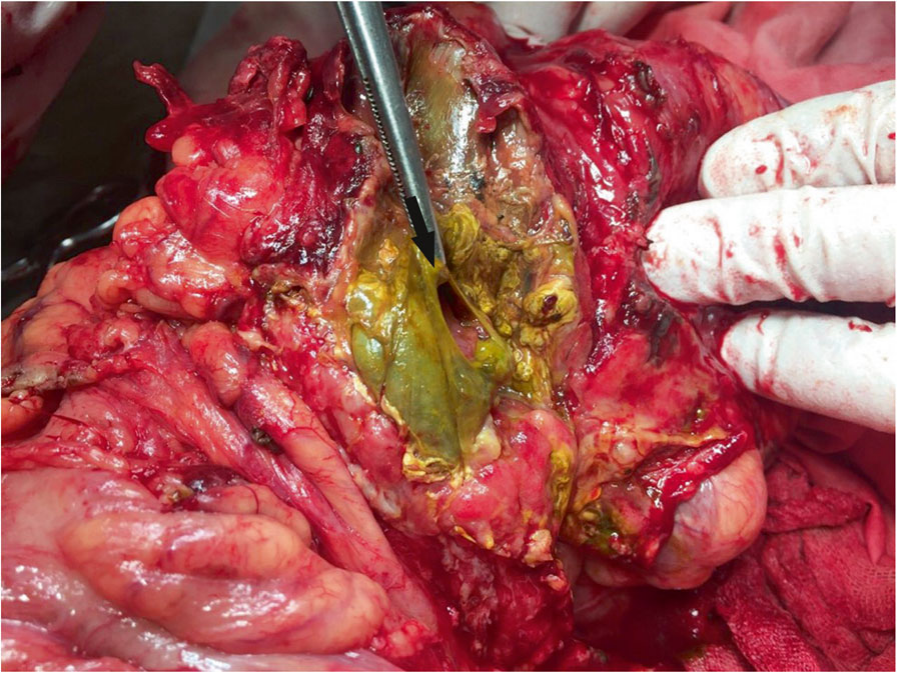
An intraoperative photograph of the transverse colon at the relaparotomy on the ninth day after the injury. A zone of the necrosis and perforation (*marked with an arrow*) at the site of previous suturing of the perforation wound of the transverse colon, fecal content of the bowel is surrounded by inflamed peritoneum

On June 7, 2017 (the ninth day after his injury), our patient was transported to the National Military Medical Clinical Center of Ministry of Defense in Kyiv in order to apply more treatment modalities. After transportation, our patient underwent relaparotomy. At revision in the right upper quadrant of his abdomen, an infiltrate was identified and dissected. While undergoing dissection, approximately 50 mL of fecal content was released and two necrotic areas (25 × 30 mm and 15 × 40 mm) with perforations were identified in the transverse colon at the site of the previous sutures (Fig. 2). Further revision revealed leakage of pancreatic juice and bile from the sutured areas of the duodenum. His duodenum was re-sutured followed by a right hemicolectomy and a side-to-side ileotransversoanastomosis. Taking into consideration the presence of local peritonitis, posttraumatic pancreatitis, and pancreatic necrosis, we decided to disconnect the stomach and the sutured zone of the duodenum from the food passage. For this purpose, an antecolic gastroenteroanastomosis with a Braun anastomosis was performed, and a nutrition tube was inserted into the efferent jejunal loop of the Braun anastomosis. Furthermore, we revealed suture insufficiency in the duodenum and leakage of pancreatic juice. Considering such intraoperative findings, we decided to perform a right hemicolectomy. We did not consider performing intestinal reconstruction by a two-stage procedure with the open abdomen because of our previous experience in the treatment of gunshot-related peritonitis after the application of high-energy weapons in patients that were injured during armed conflicts in East Ukraine. In addition, the choice of surgical approach was frequently decided intraoperatively, including in this case.

Biliary decompression was achieved by an ultrasound-guided transhepatic cholecystostomy in the right upper quadrant as an additional method for biliary decompression, and to prevent the formation of a duodenal fistula. On the second day after relaparotomy, an external duodenal fistula occurred and healed on the 29th day after the duodenum was disconnected from the food passage. On the fifth day (July 7, 2017) after relaparotomy, a suppuration of the laparotomy wound occurred. Also, a right-side upper-lobe nosocomial pneumonia and unspecified reactive hepatitis were diagnosed and treated. In the postoperative period after the relaparotomy, our patient received the following therapy: doripenem monohydrate 1.5 g/day, gatifloxacin 400 mg/day, fluconazole 100 mg/day, nadroparin calcium 2850 IU, pantoprazole 40 mg/day, epidural block, enzymes, physiotherapy, and enteral feeding.

**Fig. 3:**
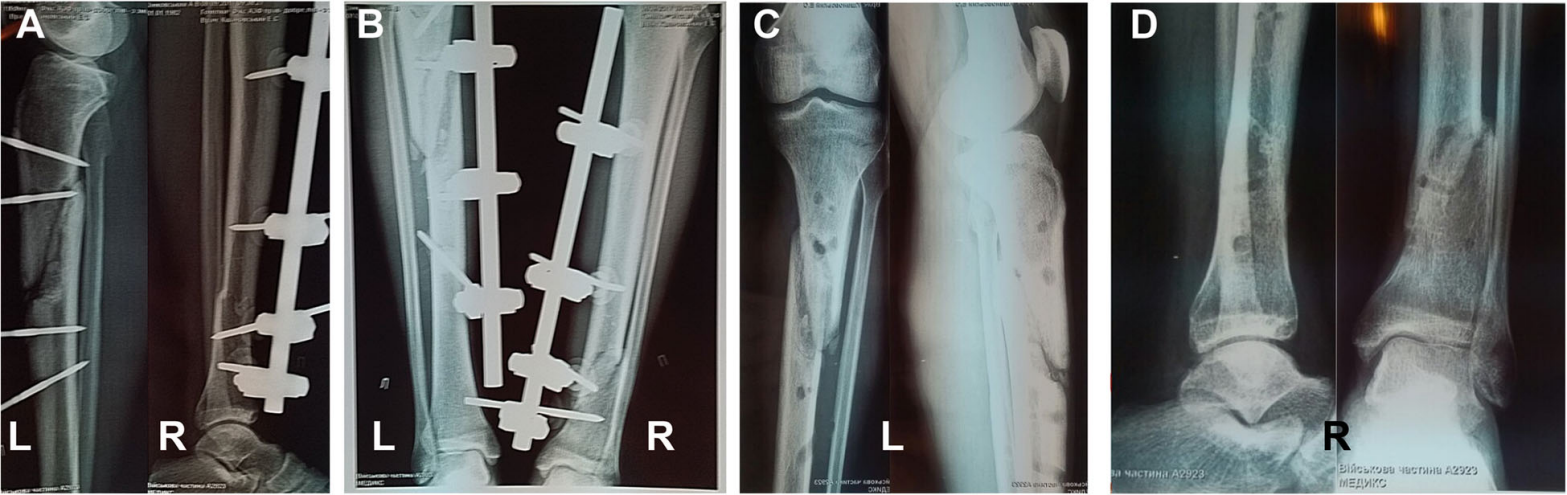
A series of the X-ray images illustrating bones fractures of the lower extremities at follow-up of the upper third of the left shin (*marked L)* and the lower third of the right shin (*marked R*), metal osteosynthesis by rod external fixation devices. **a** An X-ray image of the upper third of the left shin and the lower third of the right shin on 102nd day after the injury; **b** An X-ray image of the upper third of the left shin and the lower third of the right shin on the 154th day after the injury; **c**, **d** An X-ray image of the upper third of the lower extremities on the 168th day after the injury, the external fixation devices removed

On July 20, 2017 (the 52nd day after his injury), our patient was discharged from the surgery ward and transferred to a military rehabilitation center in the Military Medical Clinical Center of Occupational Pathology of Personnel in Irpin City. Our patient was administrated physiotherapy, exercise therapy, massage, wound redressing, symptomatic medications for the multi-fragment fractures of both shins. Bone callus formation in the fracture sites was detected (Fig. 3). Blood tests results were available at this stage (performed on August 27, 2017 – the 60th day after injury) for the following parameters: red blood cell (RBC) count of 4.6 × 10^12^/L, hemoglobin (Hb) level 127 g/L, white blood cell (WBC) count 8 × 10^9^/L, platelets 208 × 10^9^/L, erythrocyte sedimentation rate (ESR) 14 mm/h, total bilirubin 10.4 μmol/L, serum glucose 6 mmol/L, alanine transaminase (ALT) 584 U/L, aspartate transaminase (AST) 200 U/L, creatinine 89 μmol/L, total protein 66 g/L, serum urea 4.7 μmol/L, and amylase 31 U/L.

**Fig. 4:**
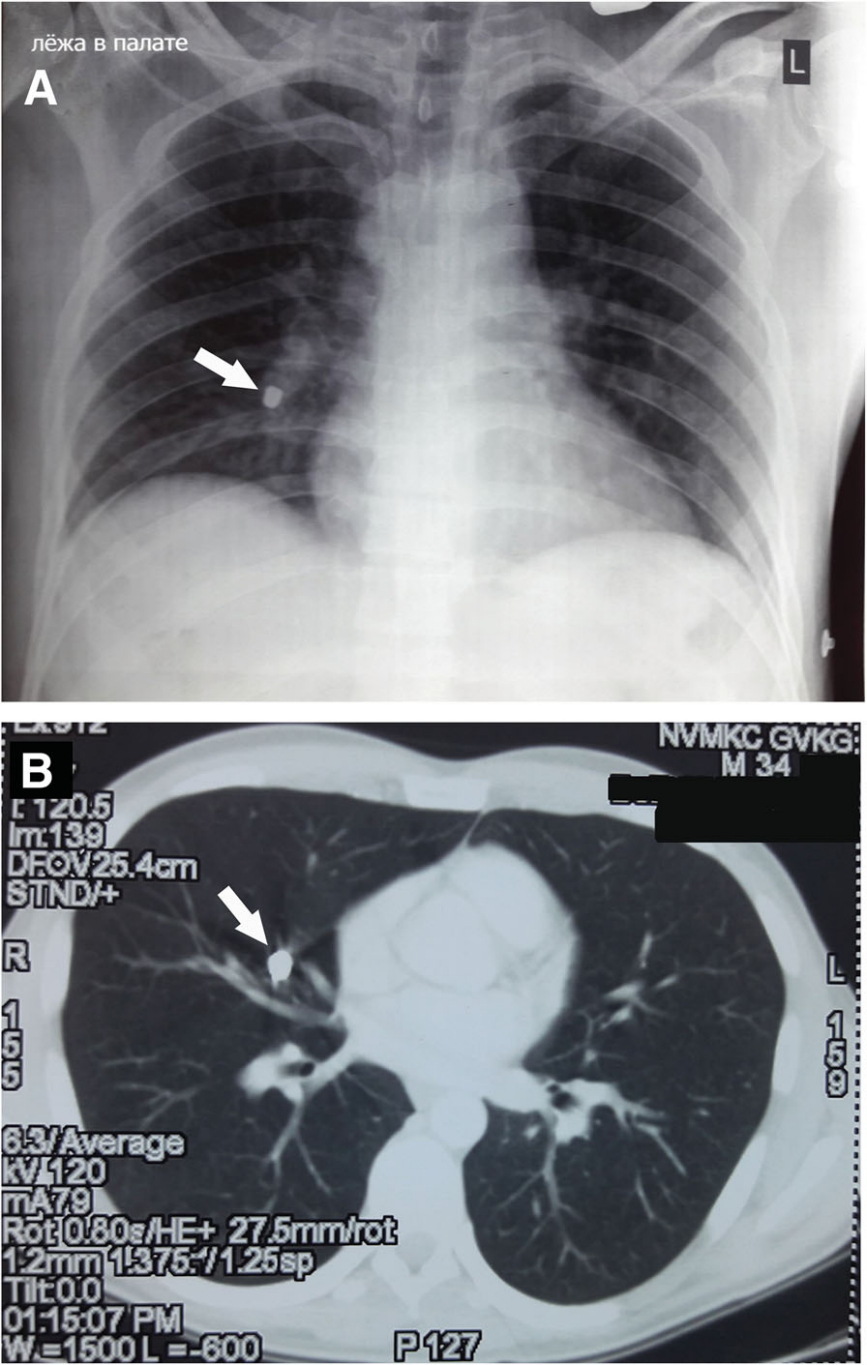
Visualization of the projectile-embolus (*marked with an arrow*) in the right mid-lobe pulmonary artery on the 63rd day after the injury by chest X-ray (**a**) and by spiral computed tomography (**b**)

**Fig. 5:**
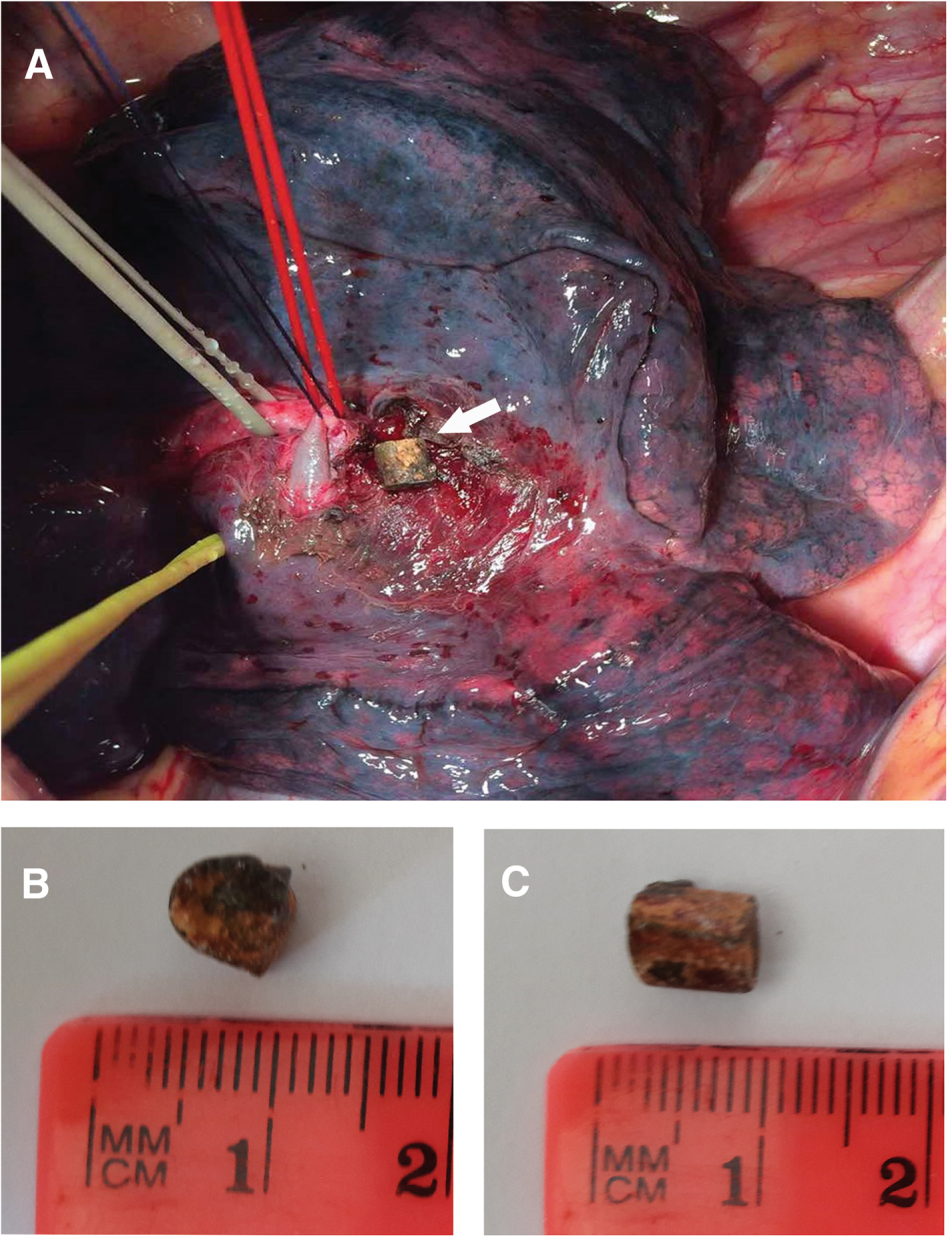
An intraoperative photograph of the thoracotomy at removal of projectile-embolus from the right mid-lobe pulmonary artery. **a** The projectile embolus is visualized as a yellowish quadrat-shaped body (*marked with an arrow*) in the lumen of pulmonary artery. The medium-lobe pulmonary vein is fixed by a yellow traction suture; the trunk of the right pulmonary artery is fixed by the white traction suture; the right mid-lobe pulmonary artery is fixed by a red traction suture; thrombosed branch of the right mid-lobe artery is fixed by a dark traction suture. Side (**b**) and front (**c**) view of the metal projectile embolus after its removal

During the 2.5 months after the injury, the fragment that migrated after the booby trap explosion in the right mid-lobe artery did not present any clinical manifestations. Meanwhile, our patient was diagnosed with bullous lung disease of the upper lobe of the right lung, thus the removal of the fragment was performed after our patient had completed the rehabilitation course. Our patient was admitted to the Department of Thoracic Surgery at the National Military Medical Clinical Center (Kyiv). A chest X-ray and CT scan showed no changes in the right mid-lobe pulmonary artery at the location site of the embolus from the booby trap fragment (Fig. 4). On August 17, 2017 (the 80th day after injury), our patient underwent a right-sided thoracotomy in the fifth intercostal space. On revision of the right lung, a firm metal object with a diameter about 10 mm was identified in the basal region of the middle lobe. The mediastinal pleura was incised, the branches of the right upper pulmonary vein were identified. The right pulmonary artery and the mid-lobe artery were taken to traction sutures (Fig. 5a). The booby trap metal fragment was identified in the lumen of the branch of the right mid-lobe artery at the medial segment, next to the bifurcation of the lobar artery. The wall of the artery was cut above the location of the metal fragment and a metal object 8 × 6 mm in diameter was visualized tightly fixed to the wall of the vessel (Fig. 5b). Analyses of the shape and kind of the embolus showed features of a piece of metal wire, which was a hand-made element that acted as additional shrapnel and was manually added to the conventional F1 hand grenade, to achieve a higher crippling effect against military personnel. Further, the distal lumen of the right mid-lobe artery was thrombosed, while the blood supply and aeration of the middle lobe of the right lung were preserved due to the collaterals. The embolus bed was washed with antiseptic, followed by ligation of the proximal and distal parts of the artery. An atypical resection of the upper lobe of the right lung was performed considering the presence of the multiple lung bullas up to 15 mm in the upper lobe of the right lung. After thoracotomy, our patient was administrated for ciprofloxacin 600 mg/day, ertapenem 1 g/day, and amikacin sulfate 1 g/day.

**Fig. 6:**
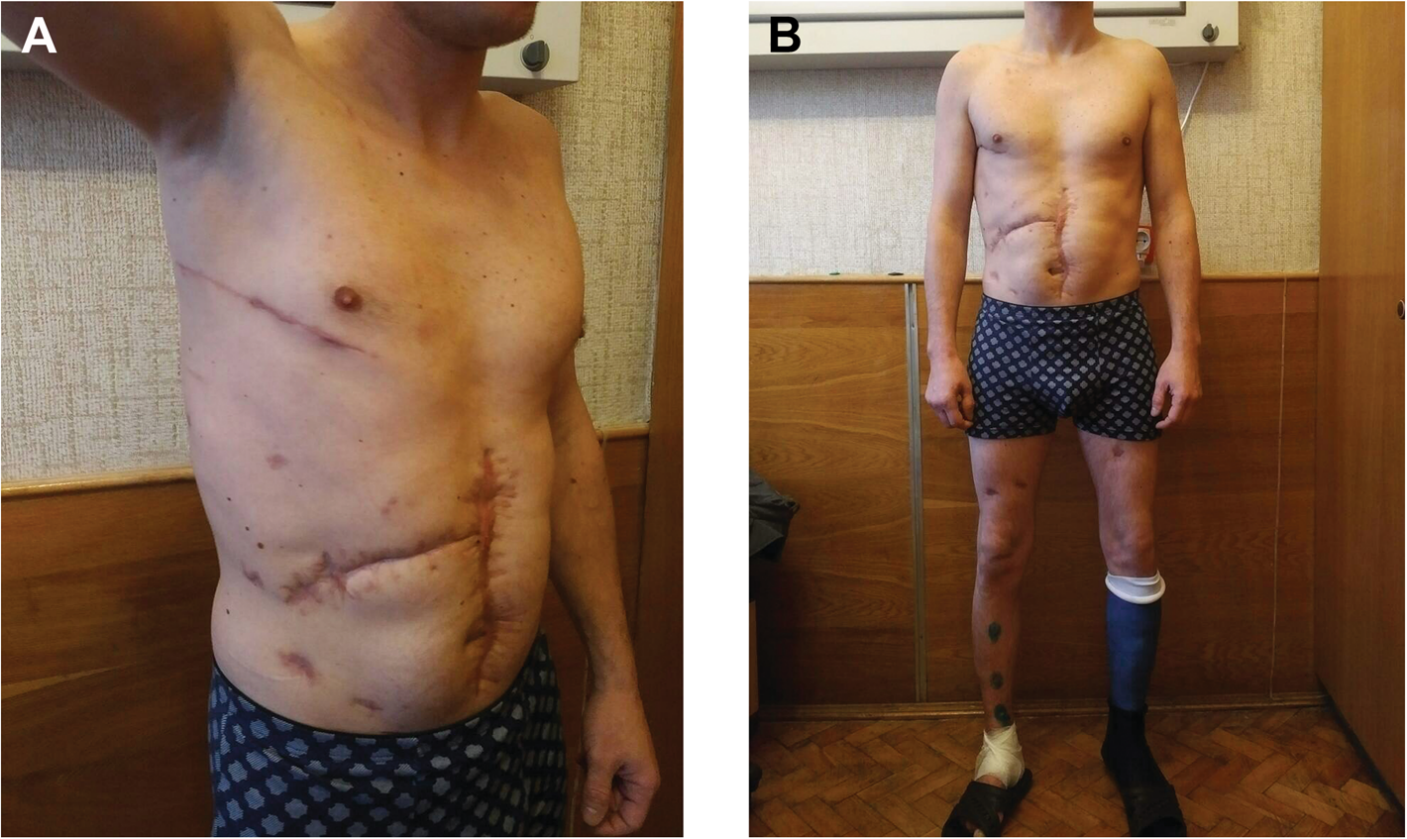
The overall look of the patient with the postoperative and post-injury scars before discharge from hospital (168th day after the injury). Anterior lateral (**a**) and front (**b**) view of the patient in the standing position

The bone fractures of both extremities were consolidated, external fixation devices were removed on the 168th day after the injury (Fig. 3c). Our patient was discharged from the hospital in a satisfactory condition and then was retired from the military service (Fig. 6).

The follow-up of our patient as well as past medical, environmental, family and employment history are not available, which is a limitation. Our patient associated himself with weak alcohol consumption and moderate smoking for 10 years, which is very common in Ukrainian population.

## Discussion

In this study we presented a case of a patient with pulmonary artery embolism in a combat patient due to migration of a metal fragment after an explosion of a booby trap hand grenade during the armed conflict in Donbas, East Ukraine. This case report demonstrates high utility application of “damage control” tactics in settings of “hybrid war” and the utility application of whole-body CT scan for all hemodynamically stable combat patients injured by high-energy weapons.

To our best of our knowledge, this is a rare case of pulmonary artery embolism associated with a booby trap hand grenade explosion.

The problem of vessel embolization due to projectile is not new. Thomas Davis reported the first case of intravascular foreign body emboli in 1834, in a 10-year-old boy, who was diagnosed with a venous embolus from a wooden projectile migrating to the right heart ventricle [7, 16, 17]. Incidence of bullet embolization is very low, with less than 200 cases since Davis’s report [6, 8, 16, 18] and constituting up to a 0.3% incidence according to the study of gunshot wounds during the Vietnam War [7, 9, 16]. Although there is a lack of data about the frequency of bullet embolization in the “hybrid war” in East Ukraine, we suspect it is similar to data shown from wars in Afghanistan and Iraq, demonstrating incidence of missile embolization after penetrating injuries in up to 1.1% of patients [9, 19, 20]. The incidence of bullet embolization in the civilian settings is unknown, but it could be higher than in combat because low-energy weapons are associated with lower kinetic energy, which is of frequent use in non-war conflicts [9].

Along with other reports, our case demonstrates the pathogenesis of bullet embolism: the explosion results in production of kinetic energy, accelerating shrapnel and fragments from the explosive device, which can penetrate tissues as well a single vessel wall. According to previously published articles, bullet embolism is commonly related to small-caliber guns [6, 12, 21, 22] and low-powered projectiles, For example, from BB guns as well as other shotgun ammunition, associated with multiple small metallic pellets [23]. Similar to other reports, we found that the following conditions increased the risk of intravascular migration of the bullet (or other projectiles): missile size and body position of the patient [7, 24], hydrostatic pressure from the blood flow and gravity [6, 21, 24], vascular anatomy [6, 12], and muscular movement [7, 21].

In the presented case report, the soldier was injured due to a booby trap explosion, affecting the inferior vena cava. The fragment from the booby trap hand grenade was a piece of a metal wire, which was manually added to the explosive device as additional shrapnel. The metal fragment had a cylindrical shape, which made it possible for it to cause a complete obstruction of the pulmonary artery branch. In our opinion, the embolism was possible in this patient because the projectile lost most of it kinetic energy while passing through the tissues of the anterior abdominal wall, the large intestine, and the duodenum. The metal fragment damaged the wall of the inferior vena cava, falling into the blood flow. Our case report does not correlate with the data from published series, in which the bloodstream did not move the projectile-embolus further than the right ventricle because of the tricuspid valve’s cusps and tendon chords. According to the published series, about half of the projectile-emboli ended up in the right ventricle, one third into the pulmonary artery or in the hepatic vein, inferior vena cava, popliteal, femoral, and common iliac veins [18, 21, 25].

Approximately 75–80% of the reported cases of projectile embolism showed arterial emboli, affecting periphery arteries, and 20–25% were venous [8, 18, 21–23]. Arterial embolization occurred mostly in the lower extremities, more commonly the left side than the right [8]. Venous embolism was associated with primary injuries of the following veins: external iliac vein [8, 26, 27], inferior vena cava [9, 10], portal vein [28], renal vein [16], femoral vein [7], right ventricle of the heart [11], cranial venous sinus [6, 29–32], subclavian vein [33], and neck veins [9, 34].

After falling into the venous blood flow the projectile-embolus may cause various types of embolism. Similar to other studies, our case demonstrates antegrade venous embolism, which is a condition when the bullet-embolus migrates with the blood flow toward the right ventricle of the heart [6, 7, 10, 27, 35]. If it passes through the tricuspid valve it gets to the pulmonary artery. Similar to other reports, in this case the projectile-embolus caused no symptoms [6, 8, 22, 36].

According to the literature, there are several kinds of embolization due to combat causalities. Results of many studies demonstrated paradoxical embolism, which is characterized by a situation when a shrapnel-embolus enters the right heart chambers, and then falls into the artery through the arteriovenous fistula or as a result of perforation of the atrioventricular septum (venous-arterial paradoxical embolism) [16, 17, 24, 25, 37–40]. The “roaming” venous embolism was shown in the studies of Fernandez-Ranvier *et al.* and Nolan *et al*. demonstrating a case of projectile-embolus to be within the venous lumen moving back and forth: first in the direction to the heart with the regular blood flow and then backward from the heart [7, 9].

In contrast to the antegrade embolism, there is a retrograde venous embolism, which occurs when a projectile-embolus moves against the blood flow under the influence of gravity into the distal direction from the location of the vein injury [22, 36, 41]. Retrograde venous embolism is very rare, diagnosed in up to 15% of patients with gunshot injuries, and the effect of gravity has been suggested to be the cause [8, 9, 22]. Furthermore, there is a tardive venous embolism, which is associated with clinical impact during the months or years after the injury [16, 22]. Acute venous embolism is developed immediately after the injury or in the first 24 hours [7–9, 27]. In contrast to the arterial or the paradoxical emboli, venous projectile embolization is asymptomatic in approximately 70% of cases, which is shown in this and other studies [6, 8, 22, 35, 36]. However, the remaining 30% of the patients with projectile vessel embolism, including pulmonary emboli, may present with clinical signs of pulmonary embolism: tachycardia, tachypnea, hypoxemia, dyspnea, chest pain, or hemoptysis [10, 12, 41, 42]. Symptomatic patients with venous embolization may be identified later, sometimes months or even years after the initial injury [18, 36, 43].

In our case report, the patient did not show any signs associated with the presence of a metal embolus in the pulmonary artery. However, according to the published series, it can be associated with various side effects in up to 25% of cases: delayed embolization to the heart or the pulmonary vessels, arrhythmia, valvular dysfunction, sepsis [8], vascular occlusion with pulmonary infarction [6, 44], pulmonary abscess, erosion in the bronchus [25], pulmonary gangrene, erosion through the arterial wall and subsequent hemorrhage [10, 17, 42], endocarditis, venous thrombosis, thrombophlebitis, and severe hypoxia [9]. Mortality associated with projectile-embolus was reported in up to 6% of patients [12, 45].

The diagnosis of projectile vessel embolism is challenging. This is a rare complication of combat injury. Therefore, it is not given much consideration when evaluating gunshot trauma in the case of abdominal perforating injuries or bleeding into the peritoneal/retroperitoneal spaces. Nevertheless, the risk of projectile vessel embolism should not be ignored [10]. Since the beginning of the warfare in East Ukraine in 2014, a whole-body CT scan has been applied for all hemodynamically stable combat patients with chest or abdominal injuries in all specialized military hospitals. In our opinion, application of a whole-body CT scan is a highly useful approach to identify unrecognized injuries, including projectile embolism. Furthermore, application of a whole-body CT scan in the setting of hybrid warfare in Ukraine is in line with other reports, suggesting the high utility of the CT scan and X-ray to identify and to evaluate projectile emboli preoperatively [6, 7, 12]. Although a CT scan is highly informative, it is not available during the “golden hour” period within combat areas in East Ukraine.

By using a CT scan and X-ray, it is possible to identify a hidden and high-risk shrapnel-emboli, such as a “roaming bullet” [9, 18, 21]. These methods are also useful for the better evaluation of combat patients with larger entrance wounds as compared to exit wounds [7], to identify projectile-embolus in patients without an exit wound, or when the localization of the projectile-embolus does not correspond to the position of the bullet wound tract [12].

Similar to Carter *et al*., the localization of the projectile fragment embolus and the clinical symptoms were considered in the management of our patient [8]. According to “damage control” tactics, we postponed the surgical removal of the metal fragment, which was also in line with other reports suggesting urgent surgery in case of life-threatening complications [6, 8, 18, 24, 25]. However, it is not in agreement with Nolan *et al.* who suggest that an arterial bullet emboli should be removed as soon as diagnosed [9]. The management of such patients is still controversial. For example, studies suggest performing embolectomy for symptomatic patients, considering the possible morbidity of up to 25% in these patients [12, 45, 46], whereas other studies offer conservative measures such as supportive care therapy [16, 47, 48]. Kortbeek *et al*. showed 14 patients out of 32 to be managed non-operatively with projectile venous embolism [47], which is in line with Nagy *et al*., recommending non-surgical management for the cases of the right ventricular bullet embolus, but with the diameter of projectile < 5 mm, firmly lodged, without evidence of arrhythmia or valvular dysfunction [24].

The endovascular removal of the projectile embolus remains controversial. We did not consider an endovascular extraction of the projectile fragment immediately after its identification by a CT scan. Furthermore, according to “damage control” tactics the severe status of our patient was taken into consideration, which required stabilization of his abdominal injuries and fixing bone fractures. During the treatment time of 2.5 months after the injury, the metal fragment was considered to be intimately fixed to the vessel wall, which was subsequently proved at open surgery (Fig. 5). Furthermore, our patient had an indication for a resection of the same lung due to the presence of a bullous lung disease with a high risk of a spontaneous pneumothorax. These considerations for the endovascular removal of the projectile embolus were in agreement with other studies, suggesting open surgery if endovascular procedures was contraindicated [7, 44]. The first study of an endovascular retrieval of a gunshot projectile-embolus was reported by Hartzler *et al.*, who used a snare device to remove a bullet from the right ventricle, which was subsequently confirmed by other studies [8, 49]. Although the number of reported cases of endovascular extraction remains low, it is suggested to be a useful approach for the treatment of symptomatic cases of pulmonary bullet emboli [7, 18, 39]. However, other studies demonstrated unsuccessful attempts of endovascular removal of a projectile-embolus from vena cava inferior or lingular segment of the left pulmonary artery [9, 27]. Some studies demonstrated extraction of the projectile embolus in patients with intracardiac localization and observation in select asymptomatic patients with pulmonary arterial emboli [9, 18].

The possibility of a minimally invasive video-assisted extraction of the projectile embolus can be considered in hospitals with appropriate facilities for such operations and a skilled surgical team. This setting was not considered in our case because the lack of experience in performing such operations, which is a limitation.

It is worth mentioning that the majority of cases of projectile venous embolism were due to single gunshot vascular injuries. We found few case reports of a venous bullet embolism causing great vessel wound and associated with a gunshot injury of internal organs [8, 9].

## Conclusions

We reported a rare case of a combat patient, who was injured in the ongoing armed conflict in East Ukraine after the explosion of a booby trap hand grenade and was diagnosed with an antegrade embolism of the branch of the right mid-lobe pulmonary artery. Our case demonstrates that “damage-control tactics” and the concept of the “golden hour” were highly effective for injured in the settings of a hybrid war.

A whole-body CT scan is an effective screening method for hemodynamically stable patients to detect asymptomatic patients with projectile-embolism in the great blood vessels.

The analyses of the available literature demonstrated significant differences of the clinical considerations for the management of patients with projectile embolism in hybrid war settings as compared with the previously described cases of combat and civil gunshot injuries.

An investigation of larger cohort of combat patients with severe injuries and projectile-embolism should be performed to develop better guidelines for these patients and to save more lives.
